# Molecular genetics of β-thalassemia: A narrative review

**DOI:** 10.1097/MD.0000000000027522

**Published:** 2021-11-12

**Authors:** Tang-Her Jaing, Tsung-Yen Chang, Shih-Hsiang Chen, Chen-Wei Lin, Yu-Chuan Wen, Chia-Chi Chiu

**Affiliations:** a Divisions of Hematology and Oncology, Department of Pediatrics, Chang Gung Children's Hospital, Chang Gung University, Taoyuan, Taiwan; b School of Medicine, College of Medicine, Chang Gung University, Taoyuan, Taiwan; c Department of Nursing, Chang Gung Memorial Hospital, Taoyuan, Taiwan.

**Keywords:** β-globin gene, β-thalassemia, genetics, molecular insights, proof-of-concept

## Abstract

β-thalassemia is a hereditary hematological disease caused by over 350 mutations in the β-globin gene (HBB). Identifying the genetic variants affecting fetal hemoglobin (HbF) production combined with the α-globin genotype provides some prediction of disease severity for β-thalassemia. However, the generation of an additive composite genetic risk score predicts prognosis, and guide management requires a larger panel of genetic modifiers yet to be discovered.

Presently, using data from prior clinical trials guides the design of further research and academic studies based on gene augmentation, while fundamental insights into globin switching and new technology developments have inspired the investigation of novel gene therapy approaches.

Genetic studies have successfully characterized the causal variants and pathways involved in HbF regulation, providing novel therapeutic targets for HbF reactivation. In addition to these HBB mutation-independent strategies involving HbF synthesis de-repression, the expanding genome editing toolkit provides increased accuracy to HBB mutation-specific strategies encompassing adult hemoglobin restoration for personalized treatment of hemoglobinopathies. Allogeneic hematopoietic stem cell transplantation was, until very recently, the curative option available for patients with transfusion-dependent β-thalassemia. Gene therapy currently represents a novel therapeutic promise after many years of extensive preclinical research to optimize gene transfer protocols.

We summarize the current state of developments in the molecular genetics of β-thalassemia over the last decade, including the mechanisms associated with ineffective erythropoiesis, which have also provided valid therapeutic targets, some of which have been shown as a proof-of-concept.

## 1 Introduction

Thalassemias are among the most common groups of recessively inherited disorders worldwide and are characterized by reduced or absent production of hemoglobin (Hb) and chronic anemia of varying severity. Hb is responsible for the binding and transport of oxygen and carbon dioxide by red blood cells and is an indispensable driver of their shape, integrity, and half-life. β-thalassemia is caused by a spectrum of mutations, resulting in a quantitative reduction of β-globin chains that are structurally normal.^[1]^ Quantitative reduction in β-globin and accumulation of α-globin chains are responsible for the pathophysiology of this disorder. Advances in understanding the underlying pathophysiology of β-thalassemia have enabled clinicians and researchers to develop novel therapeutic modalities. Heterozygotes for β-thalassemia appear to be protected from the severe effects of falciparum malaria, and natural selection has increased and maintained their gene frequencies in these malarious tropical and sub-tropical regions.^[2]^ In these prevalent regions, gene frequencies for β-thalassemia ranged between 2% and 30%. However, continued and recent population migrations also meant that β-thalassemias could be found in Northern and Western Europe and North America, making this disease a global health concern.^[3]^

Differences in the severity of the phenotype are usually related to the extent of imbalance between α- and non-α-globin chain synthesis and the predominance of the free α-chain. The first primary determinant of β-thalassemia severity is the type of β allele (β0, β+, β++), ameliorated by coinheritance of interacting α-thalassemia and coinheritance of an innate ability to increase the production of γ chains. Over the last 50 years, clinical and molecular genetic studies have demonstrated how modifier genes’ coinheritance, which alters the balance of α-like and β-like globin gene expression, may transform severe, transfusion-dependent thalassemia into relatively mild forms of anemia.^[4]^ Several algorithms have been designed to reduce the pathological imbalance of the α/β ratio using several nucleic acid-based technologies such as RNAi, lentiviral mediated gene therapy, splice-switching oligonucleotides, and gene-editing technology. These approaches aim to reduce excess free α-globin, either by reducing the α-globin chain, restoring β-globin expression, and reactivating γ-globin expression, leading to reduced disease severity, treatment necessity, treatment interval, and disease complications, thus increasing the quality of life of patients and alleviating economic burden.^[5]^

**Figure 1:**
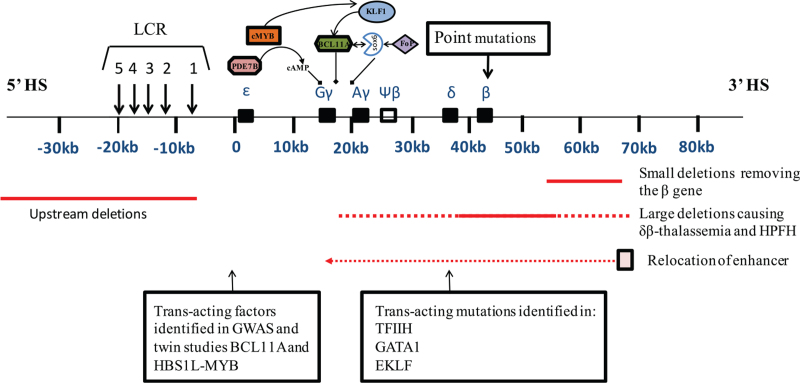
Mutations and genetic modifiers affecting β-thalassemia. The upper panel depicts the β-globin gene cluster with the upstream LCR. The mutations can be cis-acting and include point mutations affecting the structural β gene, deletions restricted to the β gene, and large deletions involving the LCR with or without the β gene. The dashed lines represent variations in the amount of flanking DNA removed by 25 different deletions, which underly δβ–thalassemia and HPFH. Transcription factors involved in the regulation of γ-globin genes are shown. A line with an arrow at the end denotes activation. A line with a black box at the end denotes repression. A dashed line denotes ambiguous interaction. A line with arrows at 2 points denotes mutual interaction.

To date, more than 350 β-thalassemia mutations have been reported in the IthaGenes database.^[6]^ Unlike α-thalassemia, in which deletions involving the α-globin gene cluster may account for the majority of the mutations, the vast spectrum of β-thalassemia mutations involved one or a limited number of nucleotides within the β-gene or its immediate flanking regions. Even though phenotype prediction from genotype is not always accurate, the information obtained from the extended genetic analysis may be used for planning appropriate management and providing adequate genetic counseling and may reveal potential new targets for therapeutic intervention. Figure 1 summarizes the mutations and genetic modifiers affecting β-thalassemia.

## 2 Methods

This narrative review was performed by collecting clinical trials, primary research, and reviews on molecular genetics and prospects for β-thalassemia therapy. Articles published in peer-reviewed scientific journals were also included. Articles were excluded if they were not written in English or published in peer-reviewed scientific journals. A waiver of authorization was obtained from our institutional review board for this comprehensive literature review.

## 3 Results

### 3.1 Genetics of β-thalassemia

β-thalassemias are heterogeneous at the molecular level. More than 350 β-thalassemia alleles have been identified, but only about 40% account for 90% or more of the β-thalassemias worldwide.^[7]^ In the areas where β-thalassemia is prevalent, only a few mutations are expected, possibly reflecting evolutionary selection by malaria. Each of these populations thus has a spectrum of β-thalassemia alleles.

The recommended molecular genetic testing approach for β-thalassemia is single-gene testing. Sequence analysis of the β-globin gene (HBB) was performed first, followed by gene-targeted deletion/duplication analysis if only one or no pathogenic variant was found.^[8]^ Analysis of HBB is complicated by the presence of highly homologous gene family members and a pseudogene, HBBP1; therefore, any assay that examines HBB sequence must be validated to ensure specificity to the active gene. In at-risk populations, targeted analysis for pathogenic variants can be performed first based on ancestry because the prevalent pathogenic variants are limited in each at-risk population.

The HBB maps clustered with other β-like genes on chromosome 11 (11p 15.15). The cluster also contains 4 other functional genes, ε-globin gene (HBE), Gγ-globin gene (HBG2), fetal Aγ(HBG1), and adult δ(HBD) as well as ψβ-pseudogene, which are arranged along the chromosome in the order of their developmental expression to produce different Hb tetramers: embryonal (Hb Gower-1 [ζ2ε2], Hb Gower-2 [α2ε2], and Hb Portland [ζ2β2], fetal (α2γ2), and adult (HbA, α2β2, and HbA2 α2δ2).^[8]^ Downregulation of HBB can be caused by a whole spectrum of molecular lesions ranging from point changes to small deletions limited to HBB to extensive deletions of the whole β globin cluster.^[9]^ In contrast to α-thalassemia, mainly caused by deletions,^[10]^ the vast majority of mutations causing β-thalassemia are non-deletional.

#### 3.1.1 Non-deletion β-thalassemia

Point mutations commonly seen in β-thalassemia are single nucleotide substitutions or oligonucleotide insertions/deletions that affect β gene expression through various mechanisms. They included single base substitutions, small insertions, or deletions of one to a few bases within the gene or its immediate flanking sequences. Point mutations affect β-globin expression in 3 different categories: mutations leading to defective β-gene transcription (promoter and 5’ untranslated region [UTR] mutations), mutations affecting messenger RNA (mRNA) processing (splice-junction and consensus sequence mutations, polyadenylation, and other 3’ UTR mutations), and mutations resulting in abnormal mRNA translation (nonsense, frameshift, and initiation codon mutations). These defects account for most β-thalassemia alleles.^[11,12]^ They downregulate HBB via almost every known stage of gene expression, from transcription to RNA processing and translation of β-globin mRNA. Approximately half of the non-deletional mutations completely inactivate the β-gene without β-globin production, resulting in β0 thalassemia.

#### 3.1.2 Deletions causing β-thalassemia

Rarely, β-thalassemia results from gross gene deletions. Deletions affecting only HBB ranged from 105 to 67 kb in size. In addition to the clinical variation of the phenotype resulting from allelic heterogeneity at the β-globin locus, the phenotype of β-thalassemia could also be modified by manipulating genetic factors mapping outside the globin gene cluster and not significantly influencing fetal hemoglobin. Two deletions remove the 3’ end but preserve the integrity of the 5’ end of the HBB. The 0.6 kb deletion involving the 3’ end of the HBB is a common cause of β0-thalassemia in Asian Indians and constitutes nearly one-third of the β-thalassemia in this population.^[13]^ The other deletions differed considerably in size but were removed in a common region in the β promoter (from 125 to + 78 relative to the mRNA cap site), including the CACCC, CCAAT, and TATA elements. They are associated with persistently high levels of HbA2 and variable increases in HbF in heterozygotes.^[1]^ Eighteen deletions restricted to HBB have been previously described. They range from 25 base pairs (bp) to ∼6 kb, of which 2 are minor intragenic deletions of 25 bp and 44 bp at the 3’ and IVSI, and 2 (619 bp and 7.7 kb) removed the 3’ end of the gene, leaving the 5’ end intact.^[14]^ In the Northwestern part of India, Sindhis and Lohanas, especially from Gujarat, show a high prevalence of the 619 bp deletion mutation,^[15,16]^ which could be iconic of Islamic conquest from the Middle East. It has been argued that the underlying mechanism for the elevated levels of HbA2 and HbF is related to deletion of the β promoter, which removes competition for the upstream β-locus control region (LCR) and “ limiting transcription factors, leading to an increased interaction of the LCR with the γ- and δ-genes in cis, thus enhancing their expression.^[2]^ This mechanism may explain the unusually high HbA2 levels that accompany point mutations in the β promoter region.^[17]^

#### 3.1.3 Dominantly inherited β-thalassemia

Dominantly inherited β-thalassemia or “inclusion body β-thalassemias” are heterogeneous at the molecular level due to mutations at or near the HBB locus. Many of these involve mutations in exon 3 of HBB. These include frameshifts, premature chain termination (nonsense) mutations, and complex rearrangements that lead to the synthesis of truncated or elongated and volatile HBBe products. The resulting β-chain variants are very unstable, and in many cases, the dominantly inherited β-thalassemia is not detectable.^[18]^ Unlike the recessive forms of β-thalassemia prevalent in malarious regions, the dominantly inherited β-thalassemia variants are rare and found in dispersed geographical regions where the gene frequency for β-thalassemia is shallow. Furthermore, many of these variants are unique to the families described and occur as de novo mutations.

#### 3.1.4 Unusual causes of β-thalassemia

Insertion of transposable elements into the IVS2 of HBB may result in approximately 15% of the normal β-globin mRNA.^[10]^ Mutations in the transcription factor TFIIH involve basal transcription and DNA repair and cause trichothiodystrophy, associated with the phenotype of β-thalassemia.^[19]^ Some mutations in the erythroid transcription factor GATA-1 have been reported to cause β-thalassemia associated with thrombocytopenia.^[20]^ Large somatic deletions at chromosome 11 p15.5, including the β-globin cluster and leading to thalassemia intermedia, have been reported in patients with heterozygous β-thalassemia. Deletion in a subpopulation of erythroid cells resulted in a somatic mosaic with 10% to 20% of erythroid cells heterozygous for one regular copy of the HBB and the remaining homozygotes without any normal HBB.

### 3.2 Genetic modifiers of β-thalassemia

As the defective genes for specific genetic disorders become unraveled, patients with almost identical genotypes may have different clinical conditions, even in simple monogenic disorders. β-thalassemia occurs when there is a deficiency in the synthesis of β-globin chains. The clinical manifestations of β-thalassemia are incredibly diverse, spanning a broad spectrum from severe anemia and transfusion dependency to the asymptomatic state of the thalassemia trait. The phenotypic diversity of β-thalassemias is prototypical of how a broad spectrum of disease severity can be generated in single-gene disorders. The most reliable and predictive factor of the disease phenotype is the mutation rate at the β-globin locus. However, the causal relationship between phenotype and genotype is further complicated by the interaction of the environment and genetic factors at the secondary and tertiary levels, some implicated in family studies and others, yet unidentified.^[21]^ Two important modifiers –coinheritance of α-thalassemia and variants associated with increased synthesis of HbF in adults, have emerged from such clinical genetic studies. Elucidation of the modifying effects of HbF and α-thalassemia has not been too difficult as these loci have an observable clinical effect and the genetic variants are common and thus would contribute significantly to disease burden. However, these 2 modifiers do not wholly explain the clinical heterogeneity.^[13,22]^ Recent advances in technology and reducing costs have prompted genome-wide association studies to derive genetic modifiers for such complex traits.^[23]^

**Figure 2:**
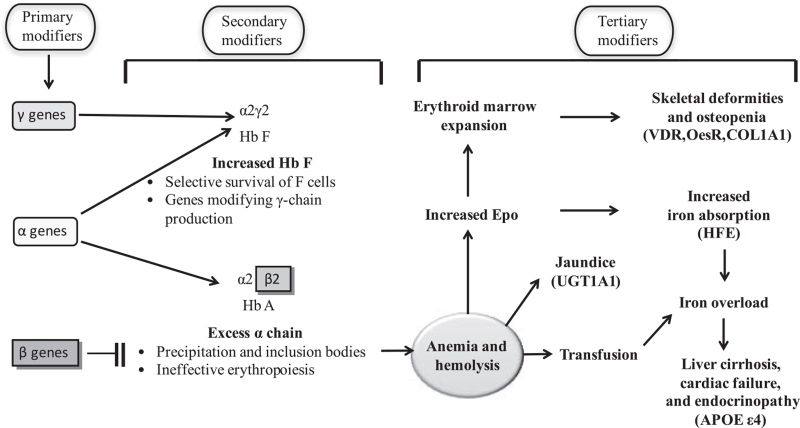
Pathophysiology of β-thalassemia. Factors that modify the β-thalassemia phenotype act at 3 levels.

However, genotypic variability at known loci is still insufficient to explain the phenotypic variability between individuals with the same genotype. A unique phenomenon known as “intra-genotypic variability” has become increasingly evident, and the relevant mechanistic underpinnings are beginning to be understood.^[24]^ This interpatient clinical variability in β-thalassemia has affected researchers’ perceptions of identifying genetic modifiers of severity for these disorders. Such genetic modifiers could lead to the development of more specific and effective therapies.^[25]^ The genetic modifiers exert their potential at 3 levels (see Fig. 2). Primary modifiers refer to the types of alterations that affect HBB. The location of mutations in different gene regions determines the associated phenotypic severity. Point mutations directly alter β-globin expression in 3 categories: mutations leading to defective HBB transcription (promoter and 5’ UTR mutations) and mutations resulting in abnormal mRNA translation (nonsense, frameshift, and initiation codon mutations). Mutations affecting transcription may result in a mild deficit in β-globin production, reflecting the relatively mild phenotype of β-thalassemia.

Secondary modifiers include the variations in genes affecting α/β globin chain equilibrium, such as α-and γ-globin genes, as well as the genes involved in γ-globin gene expression, including HBS1L-MYB, BCL11A (B-cell CLL/lymphoma 11A), KLF1 (Krüppel-like factor 1), and C1orf77, as well as gene expression affecting the amount and stability of α-globin chains. The HBS1L-MYB intragenic region (6q23) regulates erythroid cell proliferation, maturation, and HbF expression.^[26]^ It accounts for more than 20% of the variance in HbF levels in Northern Europeans. BCL11A, previously known as an oncogene involved in leukemogenesis, was discovered as an essential genetic locus regulating HbF through GWAS.^[27]^ Downstream functional studies in cell lines have shown that BCL11A represses γ-globin expression.^[28]^ KLF1 is critical in switching from γ-globin to β-globin expression; it activates HBB directly, providing a competitive edge and indirectly silencing the γ-globin genes via activation of BCL11A.^[29,30]^ C1orf77 encodes a small nuclear protein that is characterized by an arginine-and glycine-rich region. This protein may play an essential role in regulating fetal globin gene expression and activating estrogen-responsive genes.^[31]^ This globin chain imbalance can be modified by 2 factors: variation in the amount of α-globin production and variation in HbF response.

Tertiary modifiers are gene variations based on the phenotype of β-thalassemia syndrome complications. With the increasing lifespan of β-thalassemia patients, subtle variations in the phenotype concerning some complications have become more apparent, and evidence suggests that they may be affected by genetic variants. A common complication of β-thalassemia involves organ damage from iron overload, not just from blood transfusions, but also from increased absorption. In β-thalassemia patients, surveillance is needed to determine iron accumulation in the liver or to detect possible hepatocellular carcinoma growth.^[32]^

#### 3.2.1 Effect of the primary modifiers: fetal hemoglobin quantitative trait loci and α-globin genotype

In β-thalassemia, the reduced amount or absence of β-globin chains results in a relative excess of free α-globin chains in the bone marrow erythroid precursors, leading to premature death and ineffective erythropoiesis, which is the primary determinant of the severity of anemia. A minor contribution is due to peripheral hemolysis and an overall reduction in Hb synthesis. The primary variation in β-thalassemia is seen as different mutations, ranging from silent to dominantly inherited mutations. The varying degree of globin chain imbalance resulting from the variable impairment of β-globin synthesis correlates with disease severity.

Genetic studies have identified 3 major quantitative trait loci (Xmn1-HBG2, HBS1L-MYB intergenic region on chromosome 6q23, and BCL11A on chromosome 2p16) that account for 20% to 50% of the expected variation in HbF levels in patients with SCA, β-thalassemia, and healthy adults. The quantitative trait is characterized by genetic heterogeneity such that to produce a complete high-HbF phenotype, Xmn1-HBG2 must exist on a genetic background that requires the presence of additional factors. The Xmn1-HBG2 site is essential because of its considerable impact on trait variance and its high frequency (∼30%) in most population groups, including Europeans, Africans, and Asian Indians.^[33]^ Although increases in HbF and F cells associated with Xmn1-HBG2 are minimal or undetectable in healthy adults, clinical studies have shown that under conditions of stress, erythropoiesis in homozygous β-thalassemia, the presence of Xmn1-HBG2 leads to a much higher HbF response associated with a delayed transfusion need. This could explain why the same mutation on different ß-chromosomal backgrounds, some with others without the Xmn1-HBG2 variant, is associated with different clinical severities. High-resolution genotyping studies suggest that Xmn1-HBG2 may not be the causal element, but in tight linkage disequilibrium to another, as yet undiscovered, variant(s) on chromosome 11p.

#### 3.2.2 Secondary modifiers of complications of β-thalassemia

The clinical phenotype of homozygous β-thalassemia may also be modified by the coinheritance of other genetic factors mapping outside the HBB cluster and affecting some disease complications. Among these factors, the ones best delineated so far are those affecting bilirubin, iron, and bone metabolism. These modifiers do not directly affect globin imbalance but might moderate the different complications of β-thalassemia directly related to anemia or therapy, such as iron loading from intestinal absorption.^[34,35]^

Genetic modifiers can impact the phenotypical severity of β-thalassemia at the primary level by directly affecting the degree of globin chain imbalance and the moderating role of disease complications (Table 1). These include genetic variants that affect bilirubin metabolism, iron metabolism, bone disease, and cardiac complications. Jaundice and a predisposition to gallstones are associated with a polymorphic variant in the promoter of the UGT1A1 gene. Individuals homozygous for 7 [TA]s, also referred to as Gilbert's syndrome, have higher bilirubin levels and an increased tendency to form gallstones, which validated all levels of β-thalassemia.^[36]^ Several genes implicated in iron homeostasis have been characterized, including those encoding homeostatic iron regulators (HFE), transferrin receptor 2, ferroportin, hepcidin, and hemojuvelin.^[37]^ Some studies have indicated that the common mutation of the HFE gene (C282Y) causes a common type of hereditary hemochromatosis.^[38]^ The H63D variant, a common polymorphism in the HFE gene, appears to modulate iron absorption. β-thalassemia carriers who are homozygous for the HFE H63D variant have higher serum ferritin levels than carriers without the variant. The degree of iron loading, bilirubin levels, and bone mass are quantitative traits with a genetic component; variants affect the genes involved in regulating these traits, contributing to the complications.

**Table 1:** Genetic modifiers of β-thalassemia.

A. Primary at level of α/non-α-globin chain imbalance	Mechanism of action
β- globin genotype (1 or 2, severity of β-thalassemia alleles)	Directly affects output of β-globin and chain imbalance
α-globin genotype α-thalassemia co-inheritance of extra globin genes (ααα/, αααα/, or *HBA* cluster duplication)	Reduces α-globin excess Adds to redundant α-globin
Innate ability to increase HbF (co-inheritance of HbF QTLs, e.g., HbF-boosting variants in *BCL11A*, HMIP, *Xmn1-HBG2* and KLF1)	Increased γ-chains combine with excess α reducing chain imbalance
Potential modifiers include variants in ubiquitin proteolytic pathway	Promotes proteolysis of excess α-globin
α-hemoglobin stabilizing protein (AHSP)	Chaperones excess α-globin

B. Secondary at level of disease complications
Bilirubin levels and predisposition to gallstones – *UGT1A* promoter (TA)n polymorphism
Iron loading e.g., *HEF* variants
Bone disease e.g., QTLs influencing bone mass *(VDR, COL1A1, CoL1A2, TGFB1*)
Cardiac disease e.g., apolipoprotein (APOE)ε4 alleles
Predisposition to infection e.g., HLA alleles

Cardiac complications are still a leading cause of mortality and morbidity in patients with hemoglobinopathy, although heart disease due to either severe anemia or iron overload has dramatically reduced patient populations receiving modern regular therapy and follow-up. The pathophysiology of heart disease is multifactorial, reflecting iron overload, chronic anemia, and pulmonary hypertension, but genetic factors play a role. Another common complication in adults with -thalassemia is the development of marked and progressive osteoporosis, which depends on many factors, including hypogonadism and the extent of iron chelation.

#### 3.2.3 Update on the genetic control of fetal hemoglobin

Genome-wide association studies and traditional linkage studies have demonstrated several genetic loci involved in the silencing of HbF. BCL11A is a potent silencer of HbF in both mice and men. It controls the beta-globin gene cluster in concert with other factors. KLF1, a vital erythroid transcription factor, activates BCL11A and coordinates the switch from fetal to adult hemoglobin. A regulatory network of cell-intrinsic and cell-extrinsic factors maintains the epigenetic homeostasis of the beta-globin cluster and accounts for the precise lineage-specific and developmental stage-specific regulation of globin genes.^[39]^

### 3.3 Proof-of-concept therapies for β-thalassemia

Hematopoietic stem cell transplantation (HSCT) remains the definitive cure currently available for patients with β-thalassemia major.^[40]^ However, the limits of suitable donors and costs minimize their clinical application. It is accompanied by potential immune-mediated rejection and graft-versus-host disease (GVHD). There is an imperative need to explore novel therapeutic approaches – pharmacological and genetic – taking advantage of new insights into disease pathophysiology and genome technology development. Recent molecular studies of HbF regulation have reinvigorated the field and have shown promise for developing clinical HbF inducers for use in β-thalassemia patient populations.^[41]^ The new era of genome sequencing, understanding of the HBB gene cluster and its strict regulation and control, along with advancements in vector development and gene-editing platforms, has achieved greater precision and personalized treatment against β-thalassemia.

#### 3.3.1 Therapeutic induction of fetal hemoglobin

The defective production of β-globin in patients with β-thalassemia can compensate for an increase in the production of γ-globin, which pairs together with α-globin chains to form HbF. Increased production of HbF can ameliorate the severity of β-thalassemia. Consequently, there is increasing interest in the development of therapeutic approaches for inducing HbF. Inducing expression of fetal globin (γ-globin) gene expression to 60% to 70% of α-globin synthesis produces β-thalassemia trait globin synthetic ratios and has been shown to reduce anemia to mild levels that do not require regular blood transfusions. Several therapeutic classes have induced γ-globin expression in β-thalassemia patients, increased total hemoglobin levels, and even eliminated transfusion requirements in formerly transfusion-dependent patients, demonstrating the proof-of-concept of the approach. However, prior generations of therapeutics are not readily feasible for widespread use. Several recently discovered oral therapeutic candidates are currently more potent or patient-friendly, requiring low oral doses, have distinct molecular mechanisms of action, and can be used in combination regimens. Tailoring therapeutic regimens to patient subsets stratified solely for β+ or a β0 globin mutation and quantitative trait loci, which modulate HbF and clinical severity, can guide more valuable and informative clinical trials. These advancements provide a rational approach to the application of fetal globin gene induction in therapeutic regimens suitable for diverse thalassemia patient populations worldwide (Table 2).

**Table 2:** Proof-of-concept therapies for β-thalassemia.

Category	Investigational products	Mechanism of action	Reference
HbF inducer	Hydroxyurea Thalidomide, Lenalidomide, Sirolimus	Inhibition of DNA analysis Histone acetylation at γ-globin gene promotor	^[62,63]^
Activin receptor ligand traps	Luspatercept, Sotatercept	Inhibit effect of GDF-11	^[53,54]^
JAK2 inhibitor	Ruxolitinib, Pacritinib	Inhibition of signal transducer of EPO	^[64]^
Iron restriction	Hepcidin	Hepcidin binds to ferroportin, leading to its endocytosis and degradation, thus preventing the entry of iron into plasma	^[65]^
Antisense therapy	Oligonucleotide analogs	Specifically modify RNA expression through multiple mechanisms including RNase H1-mediated degradation of RNA and modulation of RNA splicing	^[66]^
Gene insertion	Vectors packaged with HBB gene and its promotor, enhancer, and parts of LCR	Insertion of a vector that contains the whole regulatory machinery and the β-globin or γ-globin producing genes into autologous HSPCs “ex-vivo,” and then infusing these modified HSPCs back to the patient after myeloablation	^[44]^
Gene editing	Different engineered nucleases-zinc-finger nucleases, transcription activator-like effector nucleases, clustered regularly interspaced short palindromic repeats (CRISPR) and CRISPR-associated-nuclease 9	nucleases that act like molecular scissors and cut the human DNA at precise locations	^[67]^

#### 3.3.2 Gene insertion and gene editing

Genetic diseases are conditions caused by one or more mutations in the genome and are ideal targets for gene therapy or gene editing, and are designed to correct the function of abnormal genes.^[42]^ Gene therapy achieves this by adding a correct copy of the gene into the genome of the cells in the target organ or tissue, while gene editing alters the genome at a specific location to correct or alter the genetic sequence.^[43]^ The premise of both these therapeutic approaches is that the presence of the modified gene enables the expression of a correctly functioning protein, eliminating the cause of the disease and improving whole-organ function.

Currently, gene therapy approaches can be divided into 2 broad groups: gene insertion and gene editing. Gene insertion is the addition of one or more genes into a DNA sequence, so that the transcription of the inserted genes can only occur in erythroid precursors.^[44]^ However, uncontrolled integration of a transgene or its regulatory sequences into undesired sites may inactivate essential genes or activate proto-oncogenes. Gene editing, namely the in situ alterations of genes by specific nucleases, represents a novel strategy due to the nuclease-associated creation of double-stranded breaks in the DNA, replacement, insertion, or deletion of a sequence in a specific locus. Such nucleases include zinc-finger nucleases, meganucleases, transcription activator-like effector nucleases, and clustered regularly interspaced short palindromic repeats (CRISPR).^[45]^

Inherited monogenic disorders, such as β-thalassemia, are candidates for gene therapy via gene transfer or gene editing.^[46]^ Ongoing technological advances in genome sequencing, stem cell selection, viral vector development, transduction, and gene editing strategies now allow for efficient ex vivo genetic manipulation of human stem cells, leading to hemoglobin production, a meaningful clinical benefit for thalassemia patients. These 3 types of gene editing technologies are expected to correct the pathogenic genes of thalassemia.^[47]^ An advanced approach for treating genetic disorders is genome editing, which utilizes targeted nucleases to correct the mutations in specific DNA sequences and restore them to the wild-type sequence. The genome-editing method can change DNA in cells or organisms to understand their physiological responses. Such genome editing tools include transcription activator-like effector nucleases, zinc-finger nucleases, and CRISPR.^[15]^ CRISPR technology is a revolutionizing treatment option for inherited diseases. This technique makes it possible to target a specific genomic locus and edit it more efficiently than possible using alternative gene-editing methods. The CRISPR/Cas system is more straightforward, rapid, safe, and efficient than the other systems. The CRISPR-associated protein 9 (CRISPR-Cas9) technique is being utilized to edit any DNA mutations associated with hereditary diseases in cells (in vitro) and animals (in vivo).^[48]^ The CRISPR/Cas9 system is expected to repair HBB in induced pluripotent stem cells, germ cells, fertilized eggs, and embryos from β-thalassemia patients, laying the foundation for future clinical applications. However, the safety profiles of such technologies remain uncertain.

Several clinical trials have investigated the safety and efficacy of gene editing to restore Hb synthesis in β-thalassemia. Most of the products are gene-addition-based techniques, while few are gene-editing strategies that aim to reactivate HbF. Although autologous HSCT using genetically corrected cells would avoid the risk of GVHD and overcome the need for a suitable donor, several requirements need to be met. These include high-efficiency gene transfer and engraftment of a high proportion of genetically modified hematopoietic stem cells (HSCs), consistent levels of HBB expression, independent of the site of integration, high expression levels of β-globin or γ-globin genes, regulated expression in the erythroid lineage, and safe expression with little or no risk of insertional mutagenesis/oncogenes.^[49]^ Since the long-term consequences of gene editing mechanisms in HSCs have not yet been clarified, current gene editing cannot be considered safer than viral-mediated gene addition.^[50]^ Various types of vectors, including viral and non-viral vectors, have been used for gene transfer. Ongoing efforts are focused on improving the efficiency of lentiviral vector-mediated gene transfer into stem cells so that the curative capacity of gene transfer can be consistently achieved.^[51]^

#### 3.3.3 Targeting ineffective erythropoiesis

##### 3.3.3.1 Activin receptor-II trap ligands

The transforming growth factor β (TGF-β) superfamily signaling acts on biological processes, such as cell quiescence, apoptosis, proliferation, differentiation, and migration, and plays an essential role in regulating hematopoiesis.^[52]^ This pathway can lose its physiological regulation in pathological conditions, leading to anemia and IE. Activin receptor-ligand trap molecules such as Sotatercept and Luspatercept downregulate the TGF-β pathway, thus inhibiting the Smad2/3 cascade and alleviating anemia in patients with β-thalassemia and myelodysplastic syndromes.^[53,54]^

##### 3.3.3.2 Iron restriction

Induction of iron restriction utilizing transferrin infusions, minihepcidins, or manipulating the hepcidin pathway prevents iron overload, redistributes iron from parenchymal cells to macrophage stores, and partially controls anemia in β-thalassemic mice. Inhibition of IE by activin ligand traps improved anemia and iron overload in the same model. Targeting iron loading or IE shows promise in preclinical studies; activin ligand traps are in clinical trials with promising results and may be helpful in patients with IE.^[55]^

The central regulator of iron homeostasis, hepcidin, is chronically repressed in this disorder, leading to unchecked intestinal iron absorption and consequent iron overload. Many groups have focused on elucidating the main pathways associated with iron regulation. New molecules have been synthesized and used in animal models of dysregulated iron metabolism, demonstrating their ability to target and reduce iron load. Antisense oligonucleotides and lipid nanoparticle (LNP)-formulated siRNAs and minihepcidin peptides are novel agents that have proven to be efficient in regulating iron metabolism in mouse models and are promising candidates for the treatment of patients with iron-related disorders.^[56]^

##### 3.3.3.3 Janus Kinase 2 inhibitors

The discovery of Janus kinase 2 (JAK2) as a vital mediator of IE and splenomegaly in β-thalassemia indicated that the application of small organic molecules to inhibit JAK2 could reduce IE and splenomegaly.^[57,58]^ Treatment of an erythropoietic disorder with an agent limiting erythropoiesis may seem counterintuitive. However, its use is rational because IE in β-thalassemia resembles leukemic cell expansion, with immature erythroid progenitors that proliferate abnormally, fail to differentiate, and invade other organs to compromise their function. Thus, the use of JAK2 inhibitors for β-thalassemia might be desirable, but it would require careful optimization, noting the potential for off-target immune suppression, as well as the anemia that would be expected from continuous JAK2 inhibition.

#### 3.3.4 Reducing α-globin synthesis

The critical pathophysiological mechanism leading to IE in β-thalassemia is the continual production of α-globin and accumulation of complementary excess α-globin in erythroid precursor cells. Over the last 30 years, clinical studies have indicated that a natural reduction in α-globin chain output through the coinheritance of α-thalassemia ameliorates the disease phenotype in patients with β-thalassemia. The challenge here is the tissue-specific selective silencing of α-globin expression to an appropriate degree to be beneficial for patients with β-thalassemia. Plausible approaches include post-transcriptional silencing through RNA interference (RNAi) using small interfering RNAs, short hairpin RNA, and epigenetic drug targeting to alter the chromatin environment genome-editing of α globin genes to disrupt the expression of α globin genes.

## 4 Discussion

There is a spectrum of phenotypes in β-thalassemia. Thalassemia genotypes can be explained by the intensities of α-/β-globin chains or α-/β-mRNA ratios. However, these are presumptive diagnoses. DNA analysis should be performed to detect the mutations. Various molecular techniques have been developed for point mutation detection in β-thalassemia and large deletion mutant breakpoints in α-thalassemia. However, these techniques have different advantages and disadvantages. Molecular screening for α- and β-thalassemia genes using next-generation sequencing has been introduced. This technique can provide an accurate diagnosis of thalassemia, and other conventional techniques may be misdiagnosed.^[15]^

However, while genotyping at the β-globin and α-globin loci is relatively easy to incorporate into the prenatal diagnosis and counseling program, detecting an inherent ability to increase Hb F response to hemopoietic stress is still difficult. Such heterocellular HPFH determinants are usually implicated in studies of family members who are currently unavailable. Before the quantitative trait loci for HbF were clearly defined, it would appear that it would be impossible to predict phenotype from genotype apart from the 2 categories of different α-globin genes with heterozygous β-thalassemia and the inheritance of mild β+ thalassemia alleles.^[59]^ Ethnicity and environment are essential factors in the analysis of genotype/phenotype relationships. Previous studies have shown that all 3 genetic modifiers – primary, secondary, and tertiary – are population-specific. The tertiary loci include many different genetic polymorphisms that form background genes, some of which have been co-selected with thalassemias. Genetic modifiers that ameliorate any secondary complications resulting from anemia or excessive iron loads due to repeated transfusions are also essential parameters that determine disease progression and severity.^[12]^

Curative treatments alter the trajectory of a patient's health care costs, from financial commitment over 50 years, into a potential “one-off” investment. Since the 1980 s, this has usually been available for only 30% of young children with matched sibling donors.^[60]^ Other novel strategies are entering clinical trials, such as erythropoiesis, through pharmacological manipulation of hepcidin and iron metabolism.

## 5 Conclusions

β-thalassemias are common disorders with a broad clinical spectrum and genetic heterogeneity. Early clinical and population studies provided evidence that an innate ability to produce fetal hemoglobin was clinically beneficial for patients with β-thalassemia and sickle cell disease, prompting many studies and clinical trials of pharmacological agents for HbF reactivation in the 1980 and 90.^[13]^ Recent progress in DNA analysis has made available a tremendous amount of information on genetic determinants that can influence the phenotype of patients and carriers. Several primary and secondary genetic modifiers have been identified, but in some cases, disease-related complications, the underlying molecular mechanism has not been elucidated to date. In 1980, allogeneic HSCT was introduced as a treatment option. To date, approximately 2000 transplants have been carried out globally. Although the results have improved dramatically through improved conditioning, time of transplant, and better support, allogeneic HSCT is limited by the availability of fully matched donors and potential immunological side effects. Gene therapy using autologous HSCs avoids the risk of GVHD and is available for all patients; however, HSCs must be genetically modified ex vivo. Thus, gene therapy for β-thalassemia has been accelerating and has reached an important crossroads of development.

Although tremendous progress has been made technically, its safety profile is still evaluated with clinical trials underway. Recent discoveries and understanding of the switch from fetal to adult hemoglobin have opened up new pharmacological and genetic targets for HbF reactivation. Similarly, an improved understanding of the mechanisms associated with ineffective and abnormal erythropoiesis has also provided valid therapeutic targets, some of which are currently being tested in clinical trials.^[61]^ A better understanding of the mechanisms of chelator action is highly desirable to improve its efficacy and reduce its additional side effects. Overall, after decades of research that included both successes and potential downfall, the path towards a permanent, donor-irrespective cure for β-thalassemia patients is steadily becoming a realistic approach.

## Acknowledgments

We would like to express our gratitude to our advisor and supervisor, Dr. Iou-Jih Hung, for guiding this work. In addition, we thank our colleagues for their assistance and constant support provided by them.

## Author contributions

**Conceptualization:** Tang-Her Jaing, Chen-Wei Lin.

**Supervision:** Tsung-Yen Chang, Shih-Hsiang Chen.

**Visualization:** Yu-Chuan Wen, Chia-Chi Chiu.

**Writing – original draft:** Tsung-Yen Chang, Chen-Wei Lin.

**Writing – review & editing:** Tang-Her Jaing, Chia-Chi Chiu.
